# Arginine and Polyamines Fate in *Leishmania* Infection

**DOI:** 10.3389/fmicb.2017.02682

**Published:** 2018-01-15

**Authors:** Sandra M. Muxel, Juliana I. Aoki, Juliane C. R. Fernandes, Maria F. Laranjeira-Silva, Ricardo A. Zampieri, Stephanie M. Acuña, Karl E. Müller, Rubia H. Vanderlinde, Lucile M. Floeter-Winter

**Affiliations:** ^1^Department of Physiology, Institute of Biosciences, University of São Paulo, São Paulo, Brazil; ^2^Department of Clinical Science, University of Bergen, Bergen, Norway

**Keywords:** promastigote, amastigote, L-arginine, ornithine, polyamine pathway, nitric oxide, arginase, nitric oxide synthase

## Abstract

*Leishmania* is a protozoan parasite that alternates its life cycle between the sand fly and the mammalian host macrophages, involving several environmental changes. The parasite responds to these changes by promoting a rapid metabolic adaptation through cellular signaling modifications that lead to transcriptional and post-transcriptional gene expression regulation and morphological modifications. Molecular approaches such as gene expression regulation, next-generation sequencing (NGS), microRNA (miRNA) expression profiling, *in cell* Western blot analyses and enzymatic activity profiling, have been used to characterize the infection of murine BALB/c and C57BL/6 macrophages, as well as the human monocytic cell-lineage THP-1, with *Leishmania amazonensis* wild type (*La-*WT) or arginase knockout (*La-*arg*^-^*). These models are being used to elucidate physiological roles of arginine and polyamines pathways and the importance of arginase for the establishment of the infection. In this review, we will describe the main aspects of *Leishmania*-host interaction, focusing on the arginine and polyamines pathways and pointing to possible targets to be used for prognosis and/or in the control of the infection. The parasite enzymes, arginase and nitric oxide synthase-like, have essential roles in the parasite survival and in the maintenance of infection. On the other hand, in mammalian macrophages, defense mechanisms are activated inducing alterations in the mRNA, miRNA and enzymatic profiles that lead to the control of infection. Furthermore, the genetic background of both parasite and host are also important to define the fate of infection.

## Introduction

The leishmaniases consist of a wide spectrum of diseases caused by more than 20 different species of the protozoa belonging to the *Leishmania* genus (Marsden, 1986; Ashford, 2000) and considered by the WHO as an important public health problem and a neglected tropical disease (WHO, 2017). The infection can generate several clinical manifestations ranging from cutaneous and mucocutaneous lesions to the lethal VL (Ashford, 2000). The disease is endemic in 98 countries around the world, with the annual incidence for CL estimated to be 0.7–1.2 million and 0.2–0.4 million for VL (Alvar et al., 2012). *Leishmania* has a dimorphic life cycle alternating between the foregut and proboscis (stomodeal valve) of the sand fly (*Phlebotomus* in Africa, Europe and Asia and *Lutzomyia* in the Americas). The replicative procyclic promastigotes form (foregut) differentiates to the infective metacyclic promastigotes and migrates to the insect stomodeal valve. The amastigote forms appear in the interior of the phagolysosomal compartment in the mammalian host macrophage (Sacks and Kamhawi, 2001; Teixeira et al., 2013).

Since the 1970’s, L-arginine has been described as an essential amino acid for *Leishmania* promastigote cultivation and growth (Krassner and Flory, 1971; Steiger and Meshnick, 1977; Steiger and Steiger, 1977). Later, L-arginine was also described as essential for *Leishmania* amastigotes survival and defining the fate of the infection in the host-parasite interaction (Wanasen et al., 2007; Wanasen and Soong, 2008). As L-arginine cannot be synthesized *de novo* (Landfear, 2011), the parasite developed complex mechanisms to take up this amino acid by specific transporters and control L-arginine availability (Darlyuk et al., 2009; Goldman-Pinkovich et al., 2016; Aoki et al., 2017a). This amino acid is the precursor in the synthesis of proteins, urea, ornithine, citrulline, nitric oxide (NO), creatinine, agmatine, glutamate, proline, putrescine, spermidine and spermine. On the other hand, L-arginine can be used to produce NO in macrophages, activate the immune response (Pekarova and Lojek, 2015) and promote the parasite killing (Roberts et al., 2004; Laranjeira-Silva and Floeter-Winter, 2014).

In this review, we will describe the main aspects of L-arginine uptake and metabolism in both parasite and host. Each section will describe the molecule that participate and modulate the main pathways where the amino acid can be found, pointing to possible targets for infection control.

## *Leishmania* L-ARGININE Uptake

*Leishmania* is auxotrophic for many amino acids, including L-arginine. Therefore, the parasite has developed a complex and specific machinery to take up the exogenous source necessary for its replication and growth (Camargo et al., 1978; Roberts et al., 2004). The control of L-arginine levels depends on the selective uptake, as well as the intracellular concentration (Vieira and Cabantchik, 1995; Vieira, 1998). Plasma membrane permeases, as well as H^+^-pumps are responsible for the arginine uptake (Zilberstein, 1993; Vieira and Cabantchik, 1995; Geraldo et al., 2005; Shaked-Mishan et al., 2006; McConville et al., 2007; Naderer and McConville, 2008; Darlyuk et al., 2009; Castilho-Martins et al., 2011). It has been shown that to control the intracellular pool of L-arginine, glutamate and methionine, *L. amazonensis* is able to sense the external availability of amino acids (Castilho-Martins et al., 2015). Another factor that can regulate the L-arginine uptake is its consumption by ARG activity. The L-arginine amount increases in the absence of ARG activity (Castilho-Martins et al., 2015). Also, intracellular amastigotes can scavenge essential amino acids from the phagolysosome (McConville et al., 2007, 2015; McConville, 2016).

Several studies have described the molecular and functional characteristics of the amino acid permeases (AAP), as a high affinity transporter of L-arginine in *L. donovani* (Zilberstein, 1993; Zilberstein and Gepstein, 1993; Kandpal et al., 1995; Akerman et al., 2004; Geraldo et al., 2005; Shaked-Mishan et al., 2006; Darlyuk et al., 2009), *L. amazonensis, L. infantum*, and *L. major* (Geraldo et al., 2005; Shaked-Mishan et al., 2006; Castilho-Martins et al., 2011; Aoki et al., 2017a). The *aap3* coding sequence is present in two copies, organized *in tandem*, in the genome of *L. donovani* (3.1 and 3.2 copies) (Shaked-Mishan et al., 2006; Darlyuk et al., 2009) and in *L. amazonensis* (5.1 and 4.7 copies) (Castilho-Martins et al., 2011). The ORF shows 98% identity between the two copies and 93% identity between *L. donovani* and *L. amazonensis* AAP3 (Shaked-Mishan et al., 2006; Castilho-Martins et al., 2011). For *L. donovani*, the 3′UTR is completely different between the two copies (Darlyuk et al., 2009). A possible explanation for two copies can be related to the post-transcriptional gene regulation according to the environmental conditions, since the two ORFs are similar. *In silico* analyses in TriTrypDB and GenBank databases has shown *aap3* orthologs in the genome of *L. infantum, L. major, L. mexicana, L. gerbilli, L. tropica, L. turanica, L. panamensis, L. braziliensis*, and *L. aethiopica* (Aoki et al., 2017a).

Ld-AAP3 mediates the uptake of L-arginine, lysine, histidine, phenylalanine and citrulline, with higher affinity for L-arginine (Kandpal et al., 1995; Shaked-Mishan et al., 2006). It does not promote the eflux of the intracellular L-arginine (Kandpal et al., 1995). The *N*-methyl + arginine acetate and phospho-L-arginine compete with L-arginine uptake indicating a stereo-specific transporter by recognition of both guanidino group and the arginine side chain, while L-arginine analogs, nitro-L-arginine methyl ester, *N*-nitro-L-arginine, aminoguanidine, agmatine and o-arginine do not modify L-arginine uptake (Kandpal et al., 1995).

Under L-arginine starvation, *L. donovani* senses the lack of the amino acid availability and responds with rapid upregulation of the expression and activity of Ld-AAP3 (Goldman-Pinkovich et al., 2016). The L-arginine starvation in *L. donovani* promastigotes, axenic amastigotes or intracellular amastigotes also lead to up-regulation of mitogen-activated protein kinase 2 (MPK2), suggesting that the parasite monitor the L-arginine availability (Goldman-Pinkovich et al., 2016). In addition, temperature change during promastigote to amastigote differentiation (25°C and 34°C, respectively) alters the amount of the *La-aap3* 5.1 transcript as well as protein expression, the localization profile in the plasma membrane and L-arginine uptake in *L. amazonensis* promastigotes. However, Aoki et al. (2017a) observed no difference in expression of the *La-aap3* 4.7 transcript as opposed to the 5.1 copy, indicating that for *L. amazonensis*, the 5.1 copy of the *aap3* gene is the one presenting the regulatory sequences for modulating the expression under different conditions (Aoki et al., 2017a).

The absence of enzymes involved in the polyamines biosynthesis, such as ARG (Castilho-Martins et al., 2015), ODC or SpdS (Darlyuk et al., 2009) reduce L-arginine uptake in *L. amazonensis* and *L. donovani.* Furthermore, the ARG absence upregulated the amino acid transporter aATP11 (Aoki et al., 2017a). Supplementation of *L. donovani* promastigote culture with putrescine reduced the L-arginine uptake, but increased the intracellular pool of L-arginine and did not affect ornithine pool. However, spermidine supplementation, reduced both L-arginine transport and the intracellular pool and ornithine levels (Darlyuk et al., 2009). The absence of ODC activity in parasites submitted to putrescine supplementation reduced the internal pool of L-arginine and maintained ornithine content, but the absence of SpdS activity reduced the internal pool of both L-arginine and ornithine (Darlyuk et al., 2009). Even with decreased L-arginine uptake, the absence of ODC and SpdS activities did not effect in abundance of the Ld-AAP3 protein (Darlyuk et al., 2009). The absence of ARG activity in *L. amazonensis* promastigotes maintained in a putrescine-containing medium lead to an increase in the internal pool of L-arginine, reduction in *La-aap3* transcripts and reduction of ornithine levels (Castilho-Martins et al., 2015).

The importance of L-arginine availability and ARG activity has also been described in intracellular amastigotes from *in vitro* infections with macrophages obtained from different organisms and genetic backgrounds, such as BALB/c and C57BL/6 mice strains and human THP-1-derived macrophages. In *Leishmania*-BALB/c and *Leishmania*-THP-1 macrophage infections, the *La-aap3 5.1* copy is upregulated during the time course of infection with *L. amazonensis* (Aoki et al., 2017a; Muxel et al., 2017). In contrast, in *Leishmania*-C57BL/6 macrophage infection, *La-aap3 5.1* is not regulated during the time course of infection with or without ARG activity (Aoki et al., 2017a). In the absence of ARG activity, both *La*-*aap3 5.1* and *4.7* copies appear up-regulated after 4 h of *Leishmania*-THP-1 infection. These differential behaviors can be explained by the distinct background of the host that can influence the L-arginine uptake and/or accumulation (Aoki et al., 2017a). Additionally, the lower availability of L-arginine in melatonin-treated BALB/c macrophage infected with *L. amazonensis*, maintained the levels of *La-aap3 5.1* and *arg* expression to keep the polyamine supply (Laranjeira-Silva et al., 2015).

To summarize, *Leishmania* senses the L-arginine availability depending on its ARG activity. The parasite also modulates the L-arginine uptake by *aap3* expression or other amino acid transporters (Goldman-Pinkovich et al., 2016; Aoki et al., 2017a). The AAP3 localization in the plasma membrane in *L. donovani* (Goldman-Pinkovich et al., 2016) and *L. amazonensis* (Aoki et al., 2017a) is directly related to this sensing mechanism. In addition, the AAP3 localization in the glycosome indicates a direct transport of the amino acid to this organelle (Hart et al., 1987; Opperdoes, 1987; Opperdoes and Szikora, 2006; Goldman-Pinkovich et al., 2016; Aoki et al., 2017a).

## *Leishmania* L-ARGININE Metabolism

Besides the L-arginine uptake, *Leishmania* also express enzymes involved in the L-arginine metabolism, including ARG and ODC, as part of the urea cycle and also, enzymes involved in the polyamines production, such as SpdS and SpmS. Since 1978, urea production has been characterized in many *Leishmania* species, and ARG activity has been highlighted as crucial for supplying ornithine for polyamines production, essential for parasite replication and the establishment of infection (Camargo et al., 1978; Mukhopadhyay and Madhubala, 1995). On the other hand, some *Leishmania* species, such as *L. braziliensis*, express other enzymes that produce urea using L-arginine, arginine deaminase and citrulline hydrolase (Yoshida and Camargo, 1978).

**FIGURE 1:**
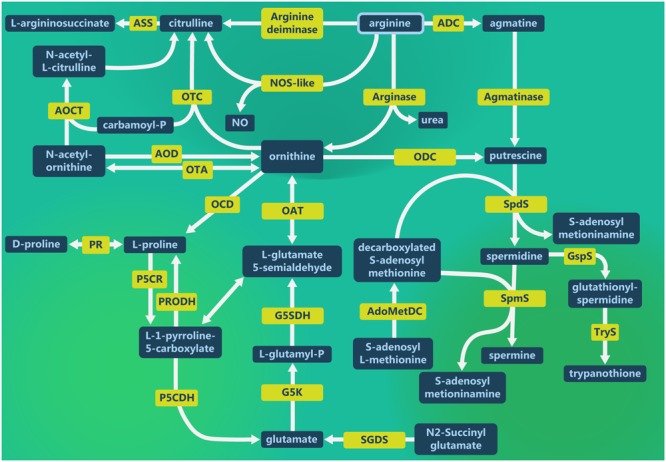
*Leishmania* L-arginine metabolism. L-arginine metabolic pathways in *Leishmania* to produce polyamines as well as the interconnection with glutamate and proline metabolism. L-arginine supplies citrulline and nitric oxide (NO) production. Ornithine supplies proline, glutamate and putrescine production, as well as the interconvertion of glutamate, proline and ornithine. Putrescine is the first polyamine that can form spermidine and spermine, essential for parasite growth. Spermidine is the substrate for spermine and trypanothione production. ADC, arginine decarboxylase; AOCT, *N*-acetylornithine carbamoyltransferase; AOD, acetylornithine deacetylase; AdoMetDC, adenosylmethionine decarboxylase; ASS, argininosuccinate synthase; G5K, glutamate 5-kinase; G5SDH, glutamate-5-semialdehyde dehydrogenase; GspS, glutathionylspermidine synthase; NOS-like, nitric oxide synthase-like; OAT, ornithine aminotransferase; OCD, ornithine cyclodeaminase; ODC, ornithine decarboxylase; OTA, ornithine transacetylase; OTC, ornithine transcarbamylase; P5CDH, pyrroline-5-carboxylate dehydrogenase; P5CR, pyrroline 5-carboxilate reductase; PRODH, proline dehydrogenase; PR, proline racemase; SGDS, succinylglutamate desuccinylase; SpdS, spermidine synthase; SpmS, spermine synthase and TryS, trypanothione synthase.

The *Leishmania* pathways involving L-arginine are demonstrated in the **Figure 1**. L-arginine is hydrolyzed by ARG to produce urea and ornithine in the first step of the polyamine pathway. Then, ornithine is decarboxylated by ODC to produce putrescine, which is substrate for spermidine and spermine production by SpdS and SpmS, respectively, adding an aminopropyl group from decarboxylated *S*-adenosylmethionine (dAdoMet) (Wu and Morris, 1998; Basselin et al., 2000; Roberts et al., 2001, 2002; Gilroy et al., 2011; da Silva et al., 2012). In addition, *Leishmania* also uses L-arginine to produce NO and citrulline (Genestra et al., 2006b; Sarkar et al., 2011; Castilho-Martins et al., 2015).

ARG is a metalloenzyme that requires two manganese atoms for enzymatic activity and uses a water molecule to attack the L-arginine substrate (Kanyo et al., 1996; da Silva et al., 2008). The ARG expression has been used as genus identification and classification tool in parasitic protozoa of Trypanosomatidae family, such as *Leishmania* genus (Camargo et al., 1978; Yoshida and Camargo, 1978). da Silva et al. (2002) described the molecular characterization of the ARG-encoding gene and its genomic organization in *L. amazonensis, L. major*, and *L. mexicana*.

ARG is compartmentalized in the glycosome of promastigotes and amastigotes, as identified for *L. amazonensis, L. mexicana* and *L. donovani* (Hart et al., 1987; Opperdoes, 1987; Roberts et al., 2004; Opperdoes and Szikora, 2006; da Silva et al., 2008; Laranjeira-Silva et al., 2012; D’Antonio et al., 2013; Boitz et al., 2017). The glycosomal localization of ARG is crucial for its activity in amastigotes of *L. amazonensis* and *L. mexicana*, since the mislocation of the enzyme reduced *in vitro* and *in vivo* infectivity, emphasizing the importance of this localization for proper ARG function (Roberts et al., 2004; Laranjeira-Silva et al., 2012; Laranjeira-Silva and Floeter-Winter, 2014; Boitz et al., 2017). In addition, *L. amazonensis* axenic amastigotes present a down-regulated ARG expression when compared to promastigotes (Aoki et al., 2017b). However, an increase of ARG expression in intracellular amastigotes during the time course of BALB/c macrophages infection was observed in *L. amazonensis* (Muxel et al., 2017). These data reinforce the existence of different modulation of ARG expression under environment conditions, such as axenic cultivation of parasites.

Metabolomic data from *L. amazonensis* promastigotes indicate that the absence of ARG activity increased the levels of the intracellular pool of L-arginine, citrulline and L-glutamate 5-semialdehyde and reduced proline, aspartate, ornithine, and putrescine, but spermidine and spermine levels were not altered (Castilho-Martins et al., 2015). RNA-seq data support the idea that by modulating the expression of enzymes involved in consumption and production of metabolites from L-arginine, *L. amazonensis* is able to surpass the absence of ARG activity (Aoki et al., 2017b).

Despite the lower levels of L-arginine, starved promastigotes increase the citrulline levels, reduce ornithine and putrescine levels and maintain the production of urea and agmatine. Ornithine can be used to produce citrulline through OTC that catalyzes the reaction between ornithine and carbamoyl phosphate (CP) (**Figure 1**). This last compound is produced from bicarbonate/ammonia and ATP (NH_3_/HCO_3_^-^/ATP) through CPS. Furthermore, a lower level of AOD transcripts in *La-*arg^-^ may reduce the conversion of *N2*-acetylornithine in ornithine (Castilho-Martins et al., 2015; Aoki et al., 2017b).

The increase in citrulline levels in *La*-arg^-^ can be due to the conversion of L-arginine in NO and citrulline via NOS-like activity (Genestra et al., 2003; Genestra et al., 2006a,b; Sarkar et al., 2009; Sarkar et al., 2011; Acuña et al., 2017) or the conversion of citrulline via arginine deaminase. There are no reports of any functional activity of *Leishmania* arginine deaminase to produce citrulline from arginine. The sustenance of high level of citrulline can occur via the reduction in transcript levels of arginosuccinate synthase which thereby reduces the conversion of citrulline into argininosuccinate (Aoki et al., 2017b). As L-arginine cannot be *de novo* synthesized in *Leishmania* (Landfear, 2011), implicating in the control of internal L-arginine availability by parasite transporters for its survival (Mandal et al., 2016). Yet, ASS activity converting citrulline to arginosuccinate has been demonstrated. It seems that ASS activity is not related with L-arginine synthesis, but with important biological functions of arginosuccinate in the establishment of the infection (Lakhal-Naouar et al., 2012; Sardar et al., 2016).

Even with the reduction in putrescine levels and SpdS transcripts, the levels of spermidine and spermine are maintained (Castilho-Martins et al., 2015). Spermine supplementation did not rescue ODC knockout *L. donovani*, and spermidine supplementation did not rescue ARG knockout *L. donovani* (Boitz et al., 2017). These observations show that interconversion of spermine to spermidine and putrescine do not occur, supporting that putrescine and spermidine are critical for the survival and the growth of the parasite (Roberts et al., 2001; Boitz et al., 2017).

Spermidine provides the production of trypanothione, an important regulator of intracellular thiol redox balance. Trypanothione also acts in the detoxification of hydroperoxide and other stressing chemical oxidant, via the enzymes glutathionylspermidine synthetase (GspS) and glutathionylspermidine-dependent trypanothione synthetase (TryS) (Tovar et al., 1998; Krauth-Siegel et al., 2003; Oza et al., 2005).

L-arginine can be used to produce agmatine through arginine descarboxylase (ADC) activity and agmatine can be converted in putrescine via agmatinase (**Figure 1**). The transcripts of ADC and agmatinase have been described in *L. mexicana* and *L. amazonensis* (Fiebig et al., 2015; Aoki et al., 2017b) and the presence of agmatine in *L. amazonensis* (Castilho-Martins et al., 2015).

L-arginine is also used by promastigotes of *L. braziliensis, L. chagasi, L. donovani, L. donovani, L. infantum, L. major, L. mexicana* and *L. panamensis* to produce NO (Sarkar et al., 2011). In *L. amazonensis* L-arginine is used to produce both NO and citrulline (Genestra et al., 2003, 2006a,b; Sarkar et al., 2011; Castilho-Martins et al., 2015). Furthermore, high levels of NO production correlate with high amounts of the infective metacyclic forms in promastigote culture of *L. amazonensis* (Genestra et al., 2006b). The differentiation to axenic amastigotes elevates the amount of NO in *L. amazonensis* compared to promastigotes (Temporal et al., 2005). Higher levels of citrulline in absence of ARG activity can be the result of the conversion of L-arginine to NO and citrulline via NOS-like activity (Genestra et al., 2006a,b; Sarkar et al., 2009; Sarkar et al., 2011; Acuña et al., 2017). NO concentration defines its biological function and in this case, NO can act in post-translational modifications, such as *S*-nitrosylation and tyrosine nitration (Colasanti et al., 2002; Kawasaki et al., 2011; Martinez-Ruiz et al., 2011; Pereira et al., 2015). It can also interfere with several biological processes, such as PKC signaling, cytochrome c regulation of caspase-cascade inactivation, protein degradation and control of the redox environment, increasing the resistance to toxicity signals into macrophage (Souza et al., 2000; Balafanova et al., 2002; Ji et al., 2006; Nakagawa et al., 2007; Abello et al., 2009; Hess and Stamler, 2012). Certainly, NO is important to metacyclogenesis and amastigote differentiation (Acuña et al., 2017) and more studies are needed to elucidate the role of NO production by promastigotes and amastigotes forms.

Altogether, the internal pool of L-arginine, ornithine, and putrescine is altered in function of external availability of L-arginine, even with higher levels of ornithine than L-arginine and putrescine. ARG and ODC activity, as well as the rate and production capacity of enzymes of the polyamines pathways are linked to the fine-tuning capacity of *Leishmania* to sense the external environment. This way, the changes from the mid-gut of sand fly to phagolysosome of mammalian host cell can be sensed by promastigotes or intracellular amastigotes allowing the maintenance of cellular homeostasis.

In addition, L-arginine starvation promotes an externalization of phospholipids that bind to Annexin-V signaling the apoptosis-like cell death in *L. donovani*, which could be blocked by L-arginine, ornithine or putrescine supplementation (Mandal et al., 2016). The change in the phospholipids externalization occurs during nutrients starvation in promastigote *in vitro* growth curve and in metacyclogenesis (Weingartner et al., 2012; dos Santos et al., 2013) influencing the *Leishmania* infectivity (de Freitas Balanco et al., 2001; van Zandbergen et al., 2006; Wanderley et al., 2009).

## *Leishmania* Proline and Glutamate Metabolism

L-arginine is one of the most versatile amino acids. It can be hydrolyzed by ARG to produce ornithine and urea, as well as its interconversion into the amino acids proline and glutamate can improve the polyamines production and enable a multi-metabolic fate during parasite growth and differentiation.

Proline and 1-pyrroline-5-carboxylate (P5C) play multifaceted roles in the cellular physiology of mammalian cells. The proline pathways, to the production of polyamines, include the interconversion between proline and P5C, by proline dehydrogenase (PRODH) and pyrroline 5-carboxylate reductase (P5CR). Proline can act as a redox shuttle being oxidized in mitochondria or, still, linked with other metabolic pathways, as pentose phosphate (O’Quinn et al., 2002; Kaul et al., 2008; Wu et al., 2008). In addition, glutamate and its metabolites, such as P5C, L-glutamyl-P and L-glutamate 5-semihaldehyde, are multipurpose molecules serving as precursors of L-proline, L-ornithine, and L-arginine. Furthermore, glutamate and its metabolites help to protect cells from nutrient depletion, oxidative stress and tumor stress (Wakabayashi et al., 1991a,b; Shanware et al., 2011).

Ornithine can form L-proline via OCD, by a NAD^+^-dependent hydride transfer reaction that culminates in ammonia elimination in mammalian cells. D-proline can be converted to L-proline by PR, a reversible reaction. L-proline form P5C by P5CR. Still, P5C can be converted in L-proline by PRODH. P5C form glutamate by P5CDH. Ornithine can be interconverted to L-glutamate-5-semialdehyde (G5S) via OAT. Additionally, glutamate form L-glutamyl-P via L-glutamate-5-semialdehyde kinase (G5K), follow by G5SDH producing G5S. And G5S can be interconvert to P5C by hydration reaction (Goodman et al., 2004; Wu et al., 2008). Castilho-Martins et al. (2015) found that amino acid starvation increased the intracellular pool of glutamate and methionine and decreased proline, ornithine and putrescine. This metabolite profile correlate with enzyme levels based on the following connections: reduction of proline due to P5C by P5CR and the increase of glutamate levels via conversion of P5C through P5CDH activity. The absence of ARG reduce the level of P5CDH transcripts and increase the amount of P5CR transcript, explaining the consumption of proline to be converted to P5C (**Figure 1**) (Castilho-Martins et al., 2015; Aoki et al., 2017a). P5C is consumed to increase the levels of L-glutamate 5-semialdehyde, as well as the L-glutamyl-phosphate conversion by G5SDH (Castilho-Martins et al., 2015).

The spermidine and spermine synthesis are not reduced in amino acid starved promastigotes. This fact could be explained by the use of proline and/or glutamate to produce P5C/G5S and subsequently ornithine, citrulline and agmatine, partially helping the production of putrescine via conversion of agmatine by agmatinase to supply spermidine and spermine. In addition, increased levels of methionine can source the *S*-adenosyl L-methionine (AdoMet) formation through methionine adenosyltransferase that uses ATP. Also, AdoMetDC produces decarboxylated S-adenosyl methionine, an aminopropyl-donor molecule used together with putrescine to form spermidine and spermine.

## *Leishmania*–Host Interaction

In mammals, L-arginine metabolism can be analyzed at the whole organism level or cellular level. Its biosynthesis can instate inflammation or immune regulation during infections or at physiological steady state, respectively (Rath et al., 2014; Ribeiro-Gomes et al., 2014). In the initial steps of *Leishmania* infection, there is neutrophil and monocyte recruitment to the affected tissue leading to macrophage differentiation days after infection. This has implications for the recognition of the parasite, opsonization, phagocytosis and consequent induction of inflammatory response with NO production, ROS and pro-inflammatory responses by the mammal cells. These actions coordinate the innate immune response and parasite killing mechanisms, as showed for *L. amazonensis, L. major* and *L. donovani* (Nathan and Shiloh, 2000; Matte et al., 2001). However, *Leishmania* can escape from the anti-leishmanicidal mechanisms of macrophages, leading to amastigote differentiation and proliferation in the phagolysosome of the macrophage (Gregory and Olivier, 2005). In this evasion, the macrophage arginase (ARG1) is activated to provide substrate for polyamine pathways (Gregory and Olivier, 2005). The parasite shares the L-arginine intracellular pool with the macrophage.

Some studies have shown the importance of the PRRs in the initiation of the innate immune response, such as TLRs, that recognizes PAMPs or DAMPs (O’Neill et al., 2013). This recognition has implications on the susceptibility or resistance to *Leishmania* infection through the role of TLRs in the activation of the phagocyte. Recognition by TLRs induced antiparasitic activity and the production of pro-inflammatory cytokines, such as IL-1, IL-6, TNF, IL-12 e IFN-γ (Akira and Sato, 2003; de Veer et al., 2003; Flandin et al., 2006; El Kasmi et al., 2008), which also could influence the rate of NOS2/ARG1 during infection.

*Leishmania* can induce adaptive immune responses via cytokines produced by T CD4^+^ lymphocytes, which can be either polarized to Th1 or Th2 phenotype. Generally, Th2 cells producing IL-4, IL-10, and IL-13 cytokines are capable of inducing ARG1 activity in murine macrophages, leading to a M2 macrophage phenotype that results in NO reduction. This leads to susceptibility to *L. major, L. amazonensis* and *L. donovani* infection, as observed in BALB/c mice infections (Nasseri and Modabber, 1979; Corraliza et al., 1995; Bacellar et al., 2000; Bhattacharyya et al., 2001; Kane and Mosser, 2001; Yang et al., 2007). On the other hand, the resistance to infection is associated with Th1 response, with the production of TNF, IL-12 and IFN-γ cytokines and induction of NOS2 expression with consequent NO production resulting in a M1 macrophage functional state, as observed in C57BL/6 mice infection (Ghalib et al., 1995; Vieira et al., 1996; Wilhelm et al., 2001; Yang et al., 2007; Ben-Othman et al., 2009; Srivastava et al., 2012). Besides, the resistance to *L. donovani* infection is related to IL-12 mediated Th1 cells activated via MyD88 (Janeway and Medzhitov, 2002; de Veer et al., 2003; Medzhitov, 2007). Also, chronic disease is associated to Th2 exacerbation in *L. amazonensis, L. major* and *L. mexicana* infection in BALB/c mice (Loeuillet et al., 2016).

In skin biopsies and plasma from patients with diffuse CL patients, higher levels of host ARG1 and ODC were detected as well as anti-inflammatory and/or suppressor factor, prostaglandin E2, IL-10 and TGF-β (Melby et al., 1994; Franca-Costa et al., 2015). Lesions of patients with mucocutaneous leishmaniasis (ML) presents higher levels of IFN-γ and lower levels of IL10R than CL patients, but similar levels of TNF-α and IL-10. The exacerbation of inflammation and cytotoxicity can promote tissue destruction. In this way, in lesions of CL patients, the increased levels of cells producing granzyme B correlates with lesion size and necrotic process (Santos et al., 2013). Some *Leishmania* molecules, such as lipophosphoglycan (LPG), seem to repress IL1-α (Frankenburg et al., 1990), IL-1-β (Hatzigeorgiou et al., 1996) and IL-12 (Carrera et al., 1996; Belkaid et al., 1998; Weinheber et al., 1998) production during infection via TLR2. Indeed, *Leishmania*-LPG regulates negatively the TLR4 signaling via SOCS-1 (Nakagawa et al., 2002) and reduces TNF by induction of repressor genes, such as SOCS-1 and SOCS-3 (de Veer et al., 2003; Kavoosi et al., 2010; Faria et al., 2012). The increase in L-arginine uptake and ARG1 activity induces polyamines production, which correlates with the increase of IL-10 and reduction of IL-12 and TNF-α levels in *L. donovani* THP-1-derived macrophages infection (Mandal et al., 2017).

## *Leishmania*–Host Interaction Targeting L-ARGININE Transport and Metabolism

**FIGURE 2:**
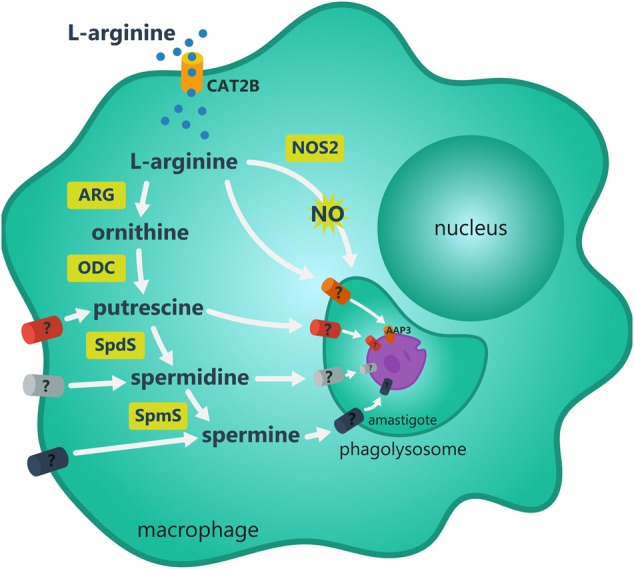
Metabolic fate of L-arginine in *Leishmania* infected macrophage. L-arginine uptake in the macrophage occurs mostly through CAT2B transporter. Once inside the macrophage, the amino acid is directed to nitric oxide (NO) or polyamines synthesis. NO is produced through NOS2 activity. Arginase (ARG) activity produces ornithine that is further decarboxylated by ornithine decarboxylase (ODC) producing putrescine, a substrate for spermidine synthase (SpdS) and spermine synthase (SpmS) producing spermidine and spermine, respectively. Some intermediate metabolites may cross phagolysossome and the parasite plasmatic membrane. These transporters have not been described yet. The L-arginine uptake occurs by amino acid permease 3 (AAP3) over the parasite plasmatic membrane and to glycosome for polyamines production by the parasite.

Controlled uptake of nutrients and ions into cells is important to maintain the intracellular homeostasis, metabolic and physiological requirements and adaptation of parasite to a hostile environment. *Leishmania* infection induces both an increase in L-arginine uptake and its hydrolysis to produce polyamines or the uptake of polyamines themselves by host cells (Bachrach et al., 1979; Schnur et al., 1979; Sacks and Kamhawi, 2001; da Silva et al., 2002, 2012; Teixeira et al., 2013; Séguin and Descoteaux, 2016), as showed in **Figure 2**. In infected macrophages, L-arginine is a common substrate for host NOS2 and ARG1 resulting in NO and citrulline production leading to parasite eradication or parasite replication via polyamine production, respectively. In addition, there are mechanisms for L-arginine uptake in both the phagolysosome and parasite membrane. Amastigotes can metabolize the amino acid to produce polyamines and/or can take up polyamines from host macrophage to replicate, also modifying the metabolism of host cell.

In mammalian cells, the endogenous synthesis of L-arginine from proline or glutamate via ornithine is not sufficient to supply the various pathways that need this amino acid as precursor, highlighting the importance of L-arginine uptake via cationic amino acid transporters (CAT1, CAT2A, CAT2B, CAT3) that differ in affinity to L-arginine, ornithine, histidine and lysine (Closs et al., 2004, 2006) Therefore, the control of L-arginine-uptake is an important factor to elicit the macrophage responses against *Leishmania* (Liew et al., 1990; Iniesta et al., 2001). Th1 cytokines production, such as TNFα and IFN-γ, induce macrophages to increase L-arginine uptake mainly via CAT2B and NO production by NOS2 leading to parasite death (Green et al., 1990a,b; Liew et al., 1991; Yeramian et al., 2006a,b; Wanasen et al., 2007). Th2 cytokines also induce CAT2B, however, ARG1 activity deviate L-arginine to polyamines production, enabling the parasite survival and replication (da Silva et al., 2002, 2012; Iniesta et al., 2002; Yeramian et al., 2006a,b; Wanasen et al., 2007).

The M1 and M2 type macrophages differ in the induction of CAT2 expression and L-arginine uptake in BALB/c and C57BL/6 mice. The natural deletion of a region in the CAT2B-coding gene promoter of C57BL/6 macrophages impairs CAT2B expression and reduces L-arginine uptake, increasing the resistance to *Leishmania* infection compared to BALB/c mice (Sans-Fons et al., 2013). *L. amazonensis* infection induces the CAT2B, CAT1 and ARG1 expression in BALB/c macrophages enabling the establishment of infection (Muxel et al., 2017). *L. major* and *L. amazonensis* repress the NOS2 expression and increase ARG1 activity, regulating the *L. major* growth via regulation of polyamines synthesis (Dixit and Mak, 2002; Kropf et al., 2005). The absence of ARG activity in *L. amazonensis* (Castilho-Martins et al., 2011; Laranjeira-Silva et al., 2012; Aoki et al., 2017b; Muxel et al., 2017), *L. donovani* (Boitz et al., 2017), *L. mexicana* (Roberts et al., 2004) and *L. major* (Roberts et al., 2004; Reguera et al., 2009) reduced the infectivity. Infection with *La-*arg^-^ reduces the levels of CAT2B, CAT1 and ARG1 and allows the expression of NOS2 and NO production in BALB/c macrophages, thereby reducing the infectivity (Laranjeira-Silva et al., 2012; Muxel et al., 2017). In *L. amazonensis* (Laranjeira-Silva et al., 2012; Muxel et al., 2017) and *L. mexicana* (Gaur et al., 2007) the absence of ARG activity reduces infectivity via NO overproduction by host macrophages, but not in *L. major* (Muleme et al., 2009). In addition, the treatment of macrophages with the hormone melatonin, blocks L-arginine uptake via reduction of CAT2B and CAT1 expression, leading to a mild reduction of ARG1 but not of NOS2 expression resulting in a reduction of infectivity of *L. amazonensis*. The importance of L-arginine in the process was shown with the recovered infectivity after putrescine supplementation of parasites treated with melatonin (Laranjeira-Silva et al., 2015). In concordance, L-arginine analog supplementation blocked the parasite growth in infected-macrophages (Iniesta et al., 2001; Wanasen et al., 2007).

CAT1 is the transporter with the highest affinity for L-arginine (Hatzoglou et al., 2004) and it is constitutively expressed in some tissues. Activation of macrophages from BALB/c with M-CSF, LPS, IFN-γ, IL-10 and IL-4, as well as L-arginine deprivation, does not modify CAT1 expression (Yeramian et al., 2006a,b). Despite this, starvation of mammalian cells increases the stability and translation of *Cat1* mRNA (Aulak et al., 1999; Hatzoglou et al., 2004; Bhattacharyya et al., 2006; Lopez et al., 2007).

Some studies have shown that the CAT2 expression in BALB/c macrophages is independent of L-arginine availability and ARG1 or NOS2 activity (Hammermann et al., 2000; Yeramian et al., 2006b). In contrast, CAT2 expression and L-arginine uptake in THP-1-derived macrophages and human monocyte-derived macrophages are dependent on L-arginine availability and host arginase activity (Mandal et al., 2017). In addition, the blocking of CAT2 reduces the L-arginine uptake in *L. donovani*-infected macrophages leading to ARG regulation and decrease of ornithine, spermidine, citrulline and NO levels (Yeramian et al., 2006a; Mandal et al., 2017). Indeed, signals for activation and proliferation of macrophages increase CAT2 levels and L-arginine uptake (Yeramian et al., 2006b). Despite this, spermidine and spermine suppress CAT2B and NOS2 levels and L-arginine uptake in rat alveolar macrophages (Mossner et al., 2001) and inhibit NOS2 activity in J774 macrophages (Szabo et al., 1994a,b). Cells with high ARG1 activity induce the uptake of large amount of L-arginine via CAT2. This may mediate the exchange of ornithine via CAT2 triggering/supplementing the neighboring cells (Closs et al., 2006). Spermine inhibits the synthesis of IL-1, TNF-α, IL-6, MIP-1α and MIP-1β in LPS-stimulated human peripheral blood mononuclear cells, which is restored by the polyamine analog 1-4-bis 3-aminopropyl-piperazine (BAP) (Zhang et al., 1997, 1999).

Functional characterization of *Leishmania* ARG, using knockout parasites revealed that the enzyme is essential for *in vitro* and *in vivo* proliferation of *L. amazonensis* (da Silva et al., 2002; Castilho-Martins et al., 2011; Laranjeira-Silva et al., 2012), *L. mexicana* (Roberts et al., 2004), *L. major* (Roberts et al., 2004; Reguera et al., 2009) and *L. donovani* (Boitz et al., 2017). Arginase null *L. amazonensis* (da Silva et al., 2002; Castilho-Martins et al., 2011; Laranjeira-Silva et al., 2012), *L. mexicana* (Roberts et al., 2004) and *L. major* (Roberts et al., 2004; Reguera et al., 2009) presented lower levels of infectivity, with exception of parasite burden in spleen of *L. donovani* infected mice (Boitz et al., 2017), highlighting differences in ARG role in the infection fate of distinct species.

## Evasion of Immune Response Pointing L-ARGININE Metabolism

Since polyamines are essential for *Leishmania* growth and NO plays an antiparasitic effect, the activity of ARG1 *versus* NOS2 is a central factor in the immune response leading to host susceptibility or resistance. These enzymes are mutually regulated due to substrate sharing transcriptional and post-transcriptional mechanisms controlling enzyme expression and activity. *Leishmania* parasites are believed to escape from the macrophage killing response by targeting the L-arginine metabolism, although the molecular mechanisms are not totally understood.

The antiparasitic activity of macrophages could be reduced via inhibition of cytokines and NO, which production is mediated by transcription factors such as NF-κB, AP-1, STAT and CREB, and reduction in PKC activity, calcium influx and ERK dephosphorylation (Ghosh et al., 2001, 2002; Forget et al., 2005a,b; Gregory and Olivier, 2005; Olivier et al., 2005; Ben-Othman et al., 2009). *L. major* and *L. amazonensis* amastigotes block the nuclear translocation of NF-κB dimer p50/p65, but increases the dimer p50/p50 translocation, which repress the transcription of pro-inflammatory cytokines (Guizani-Tabbane et al., 2004; Calegari-Silva et al., 2009), NOS2 and CAT2 (Dixit and Mak, 2002) and induces the transcription of IL-10 (Guizani-Tabbane et al., 2004; Calegari-Silva et al., 2009).

Post-transcriptional regulation by miRNAs has been recently described as an important component of the polyamines/NO pathways modulation in infected macrophages. MicroRNAs are small non-coding RNAs that are capable of binding to complementary 3′ UTR regions of target mRNA, regulating its stability or translation (Bagga et al., 2005; Lim et al., 2005). *L. amazonensis* infection increased the expression of some macrophage miRNAs, such as miR-294 and miR-721. These miRNAs interact with 3′ UTR region of *Nos2* mRNA, reducing the enzyme and NO production. Meanwhile, parasites with no ARG activity reduce the expression of those miRNAs and increased NOS2 and NO production, leading to the control of the infection of BALB/c macrophages (Muxel et al., 2017). *L. donovani* target Dicer1 (RNAse II member family), that could result in post-transcriptional regulation of host mRNAs/miRNA interactions (Ghosh et al., 2013); *Leishmania*-gp63 cleaved Dicer1 impairing the pre-miR122 processing and maturation to miR-122 preventing the binding to RNA-induced silencing complex (RISC), which guides the interaction with target mRNA and leads to gene expression regulation (Bernstein et al., 2001; Schwarz et al., 2003; Vaucheret et al., 2004; Wang et al., 2009; Ghosh et al., 2013). Furthermore, miR-122 can interact with *Cat1* mRNA 3′UTR regulating its stability and protein levels in stress conditions (Bhattacharyya et al., 2006).

## Treatments Targeting L-ARGININE Metabolism

The leishmaniases are neglected tropical diseases affecting primarily underdeveloped regions of the world. They are relatively unattractive for research development, pharmaceutical industry and financial funding. The first-line drug for leishmaniasis treatment recommended by WHO is based on pentavalent antimonial and was described by Vianna (1912). The leishmaniases chemotherapy is complicated because most of the drugs used are expensive, toxic and require long periods of supervised therapy (Murray et al., 2005; Hotez et al., 2008). The pentavalent antimonial (Glucantime and Pentostan) present several side effects and reports of parasite resistance have been described worldwide (Murray et al., 2005). Cases that are unresponsive to antimonial treatment, or patients from Europe or North America are usually treated with amphotericin or PEN, although these drugs also have several side effects (Murray et al., 2005; Croft et al., 2006). The mechanism of action of pentamidine has been related to the disintegration of the kinetoplast and mitochondria, and a collapse in mitochondrial membrane (Croft and Brazil, 1982; Vercesi and Docampo, 1992; Basselin et al., 2002). In *L. donovani*, pentamidine has been described as a competitive inhibitor of arginine transport (Kandpal et al., 1995) and a non-competitive inhibitor of putrescine and spermidine transport in *L. infantum* (Reguera et al., 1994), *L. donovani* and *L. mexicana* (Basselin et al., 2000). In addition, other compounds have been used for leishmaniasis treatment. Miltefosine was the first effective oral drug developed to treat VL. It has been used in India for decades (Jha et al., 1999), however, an increase in the failure rate has been reported (Sundar et al., 2012; Rijal et al., 2013), possibly by the selection of resistant parasites. Amphotericin B, particularly the liposomal formulation, is also an alternative line for the antimonial treatment and it is associated to binding to the membrane sterol group of *Leishmania* (Berman et al., 1998; Croft et al., 2006).

Considering the limitations of the currently used chemotherapy and the lack of effective vaccines for the leishmaniases, the identification of new drugs and vaccine approaches for the treatment of leishmaniases is required. A rational strategy for the parasite control can be developed based on the identification of fundamental metabolic pathways of the parasite. New potential drug targets based on molecular and biochemical studies involving the following: protein kinases (Naula et al., 2005), glycolytic enzymes (Chawla et al., 2010), sterol synthesis (Urbina et al., 2002; Lorente et al., 2004), purine salvage pathway (Ullman and Carter, 1995; Landfear et al., 2004; Aoki et al., 2009, 2016) and polyamine pathway (Roberts et al., 2004; Reguera et al., 2005) have been described and could be used in future therapies (Sharlow et al., 2009; Siqueira-Neto et al., 2010).

The polyamines pathway could be a promising target, since parasites need the polyamine pathway to replicate and establish the infection in the mammal host (da Silva et al., 2012). Previous description targeting polyamine-based treatment with l-α-difluoromethylornithine (DFMO) leading to the ODC inhibition in *T. brucei* is described successful against African sleeping sickness (Van Nieuwenhove et al., 1985). However, the efficacy of DFMO for *Leishmania* is controversial. Previous studies demonstrate the efficacy for *L. donovani, L. infantum* and *L. guyanensis* infections but not for *L. major* and *L. mexicana* (Gradoni et al., 1989; Keithly and Fairlamb, 1989). Furthermore, other studies describe the DFMO inefficacy against *Leishmania* (Kaur et al., 1986).

Other ODC inhibitors have been described, such as 3-aminooxy-1-aminopropane (APA), an isosteric analog of putrescine, effective in *L. donovani* promastigotes and amastigotes (Singh et al., 2007). *L. donovani* AdoMetDC was the target for A5′-((Z)-4-amino-2-butenyl)methylamino)-5′-deoxyadenosine (MDL73811) and overexpression of AdoMetDC in *L. donovani* promastigotes conferred resistance to MDL73811, but not to pentamidine, berenil and mitoguazone (MGBG), inhibitors of dAdoMet activity *in vitro* (Roberts et al., 2002). The overexpression of ODC, AdoMetDC and SpdS in *L. donovani* promastigotes conferred resistance to DFMO, MDL73811, and *n*-butylamine (Roberts et al., 2007). Interestingly, the resistance to antimonial drugs correlates with ODC overexpression in *L. tarentolae* (Haimeur et al., 1999). Nitric oxide synthase inhibitor L-NGmonomethylarginine (L-NMMA) reduce the levels of NO and increase the infectivity of *L. infantum* in human macrophages (Panaro et al., 2001). Controversially, some polyamines analogs or inhibitors could be cytotoxic for host cells because of blocking the polyamines production. In this way, further studies must explore a specific drug for polyamines enzymes of the parasite.

Gene knockout experiments have also been used to demonstrate the importance of some genes in the polyamine pathway as essential for parasite growth (Laranjeira-Silva et al., 2012; Laranjeira-Silva and Floeter-Winter, 2014). ARG, ODC, AdoMetDC or SpdS null mutant knockout lines have been providing important data on the dependence on supplementation with the substrates for parasite replication in *L. donovani, L. major, L. mexicana* and *L. amazonensis* (Laranjeira-Silva et al., 2012; Laranjeira-Silva and Floeter-Winter, 2014; Boitz et al., 2017; Aoki et al., 2017b; Roberts and Ullman, 2017). In addition, *in vivo* and *in vitro* infections present lower infection index, demonstrating how this pathway can a promisor target chemotherapy.

Another fact that may be considered for new treatment strategy is the fundamental differences between the parasite and its host. A promising target can be the amino acids uptake. As mentioned before, L-arginine uptake in macrophages is mediated by CAT (Closs et al., 2004). In contrast, *Leishmania* has a complex and specific machinery to take up this amino acid. L-arginine uptake in *Leishmania* is mediated by AAP3 (Darlyuk et al., 2009; Castilho-Martins et al., 2011; Aoki et al., 2017a). Since ARG and AAP3 are localized in the plasma membrane and compartmentalized in the glycosome of promastigotes and axenic amastigotes (da Silva et al., 2012; Aoki et al., 2017a), a strategy to control *Leishmania* infection could be focused on the inhibition of L-arginine flux by both plasmatic membrane and glycosome of the parasite.

## Concluding Remarks

In this review, we elucidate the arginine and polyamine in *Leishmania* infection by both host and parasite. The amino acid availability defines the fate of infection for immune response control or for parasite replication. Activated macrophages produce leishmanicidal molecules, such as NO, that lead to parasite killing, whereas induce ARG expression to parasite replication. These two fates of infection use L-arginine as a common substrate for their enzymatic activities.

## Author Contributions

SM, JA, JF, and LF-W wrote the original article. JF designed the figures. SM, JA, JF, RZ, SA, KM, RV, LF-W, and ML-S revised the article.

## Conflict of Interest Statement

The authors declare that the research was conducted in the absence of any commercial or financial relationships that could be construed as a potential conflict of interest.

## Abbreviations

AAP3: amino acid permease 3
ADC: arginine decarboxylase
AdoMetDC: adenosylmethionine decarboxylase
AOCT: *N*-acetylornithine carbamoyltransferase
AOD: acetylornithine deacetylase
ARG: arginase
ASL: argininosuccinate lyase
ASS: argininosuccinate synthase
CAT: cationic amino acid transporter
CL: cutaneous leishmaniasis
CP: carbamoyl phosphate
CPS: carbamoyl phosphate synthetase
DAMP: danger-associated molecular pattern
DFMO: difluoromethylornithine
G5K: glutamate 5-kinase
G5SDH: glutamate-5-semialdehyde dehydrogenase
GspS: glutathionylspermidine synthase
La: *L. amazonensis*
Ld: *L. donovani*
IFN: interferon
IL: interleukin
ML: mucosal leishmaniasis
MPK2: mitogen protein kinase 2
mRNA: messenger RNA
miRNA: microRNA
NGS: next-generation sequencing
NO: nitric oxide
NOS: nitric oxide synthase
OAT: ornithine aminotransferase
OCD: ornithine cyclodeaminase
ODC: ornithine decarboxylase
ORF: open reading frame
OTA: ornithine transacetylase
OTC: ornithine transcarbamylase
P5C: pyrroline-5-carboxylate
P5CDH: pyrroline-5-carboxylate dehydrogenase
P5CR: pyrroline-5-carboxylate reductase
PAMP: pathogen-associated molecular pattern
PD: proline dehydrogenase
PEN: pentamidine
PR: proline racemase
PRR: pattern recognition receptor
ROS: reactive oxygen species
SGDS: succinylglutamate desuccinylase
SpdS: spermidine synthase
SpmS: spermine synthase
TLR: toll-like receptor
TNF: tumor necrosis factor
TryS: trypanothione synthase
UTR: untranslated region
VL: visceral leishmaniasis
WHO: World Health Organization
